# Increased serum levels of advanced glycation end products are negatively associated with relative muscle strength in patients with type 2 diabetes mellitus

**DOI:** 10.1186/s12902-022-01035-1

**Published:** 2022-05-04

**Authors:** Tsung-Hui Wu, Shiow-Chwen Tsai, Hsuan-Wei Lin, Chiao-Nan Chen, Chii-Min Hwu

**Affiliations:** 1 Section of Endocrinology and Metabolism, Department of Medicine, Taipei Veterans General Hospital, 201 Shi-Pai Rd. Sec. 2, Chung-Cheng Build. 11F Room 522, Taipei, 112 Taiwan; 2 Institute of Sports Science, University of Taipei, Taipei, Taiwan; 3 Section of Endocrinology and Metabolism, Department of Internal Medicine, Taipei Veterans General Hospital, Hsinchu Branch, Hsinchu, Taiwan; 4 Department of Physical Therapy and Assistive Technology, School of Biomedical Science and Engineering, National Yang Ming Chiao Tung University, Taipei, Taiwan; 5 Faculty of Medicine, National Yang Ming Chiao Tung University School of Medicine, Taipei, Taiwan

**Keywords:** Advanced glycation end products, Relative muscle strength, Diabetic peripheral neuropathy, Type 2 diabetes mellitus

## Abstract

**Background:**

In this study, we investigated whether serum levels of advanced glycation end products (AGEs) independently correlated with relative muscle strength after adjustment for clinical variables including diabetic peripheral neuropathy in patients with type 2 diabetes. Relative muscle strength was defined as muscle strength (in decinewtons, dN) divided by total muscle mass (in kg).

**Methods:**

We enrolled 152 ambulatory patients with type 2 diabetes. Each participant underwent measurements of muscle strength and total muscle mass. Serum levels of AGEs were determined. The Michigan Neuropathy Screening Instrument Physical Examination (MNSI-PE) was performed to assess diabetic peripheral neuropathy.

**Results:**

The participants were divided into three groups on the basis of tertiles of serum AGEs levels. Significant differences were observed among the three groups in relative handgrip strength (71.03 ± 18.22, 63.17 ± 13.82, and 61.47 ± 13.95 dN/kg in the low-tertile, mid-tertile, and high-tertile groups, respectively, *P* = 0.005). The relative muscle strength of the ankle plantar flexors was higher in the low-tertile group than in the mid-tertile and high-tertile groups (107.60 ± 26.53, 94.97 ± 19.72, and 94.18 ± 16.09 dN/kg in the low-tertile, mid-tertile, and high-tertile groups, respectively, *P* = 0.002). After adjustment for MNSI-PE score and other clinical variables in partial correlation analysis, the correlations between serum levels of AGEs and the relative muscle strength of handgrip, ankle dorsiflexor, and ankle plantar flexor remained significant. Serum AGEs level was the only variable that remained significantly related to the relative muscle strength of handgrip, ankle dorsiflexor, and ankle plantar flexor when AGEs level, fasting plasma glucose, and glycated hemoglobin (HbA_1c_) were entered into multiple regression models simultaneously.

**Conclusions:**

After adjustment for multiple factors including diabetic peripheral neuropathy, this study demonstrated that increased serum levels of AGEs were independently associated with decreased relative muscle strength in patients with type 2 diabetes. Compared with fasting plasma glucose and HbA_1c_, serum level of AGEs is more strongly associated with relative muscle strength.

## Background

Although loss of muscle strength is related to the aging process, an accelerated decline in muscle strength has been described in patients with diabetes mellitus [1]. A longitudinal analysis from the Health, Aging, and Body Composition Study demonstrated that older patients with diabetes had a steeper muscle strength decline over time compared with control individuals without diabetes [2]. A progressive loss of muscle strength increases the risks of functional dependency and disability in patients with type 2 diabetes [3]. Moreover, poor muscle strength in these patients is associated with hospitalization, cardiovascular events, and mortality [4].

Muscle impairment in patients with diabetes has been attributed to insulin resistance, hyperglycemia, muscle fat infiltration, chronic inflammation and oxidative stress, peripheral arterial disease, and peripheral neuropathy [5]. Recently, researchers proposed that advanced glycation end products (AGEs) could play a crucial role in the pathogenesis of diabetic muscular dysfunction [6].

AGEs are formed through the nonenzymatic reactions of reducing sugars and their metabolites with lipids, proteins, and nucleic acids [7]. Hyperglycemia may cause up-regulation of the polyol and hexosamine pathways, both of which increase endogenous AGEs production [8]. Mechanisms mediating the detrimental effects of AGEs include protein structures modification, AGEs receptor-mediated oxidative processes, and intracellular accumulation of AGEs [9, 10]. Evidence indicates that AGEs may contribute to a progressive and generalized loss of muscle strength [11]. Older community-dwelling adults with elevated serum carboxymethyl-lysine, a major AGE, have greater risks of poor grip strength and low walking speed [12, 13].

However, studies have reported that the formation and accumulation of AGEs in peripheral nerves are involved in the development of diabetic neuropathy [14]. Peripheral neuropathy is another cause of muscle strength decline in patients with diabetes [11]. Whether the association between AGEs and muscle strength decline in patients with type 2 diabetes is independent of diabetic peripheral neuropathy has not been comprehensively investigated.

Therefore, the purpose of the study was to examine the relationship between serum levels of AGEs and muscle strength in patients with type 2 diabetes while carefully considering the potential confounding influence of peripheral neuropathy. We hypothesized that high serum levels of AGEs are negatively associated with muscle strength in patients with type 2 diabetes after adjustment for diabetic peripheral neuropathy.

## Methods

### Patients

As participants in this study, we recruited ambulatory patients who were aged 50 years or older and who had type 2 diabetes mellitus for at least 1 year from the diabetes clinics at Taipei Veterans General Hospital. Patients with an estimated glomerular filtration rate of less than 15 mL/min/1.73m^2^ (as per the Modification of Diet in Renal Disease Study equation) or with a history of severe congestive heart failure (New York Heart Association Functional Class III or IV), severe stroke (National Institutes of Health Stroke Scale score > 15), peripheral arterial occlusive disease, or severe muscular disorders were excluded. Patients were also excluded if they had been diagnosed with foot ulcers or non-diabetic neuropathy within the previous 6 months. This study was approved by the ethics committee of Taipei Veterans General Hospital. Written informed consent was obtained from each participant prior to their enrollment in the study.

### Measurements

Eligible participants were scheduled for anthropometric and blood pressure measurements at 8 a.m. after a 12-h overnight fast. Seated blood pressure was measured using an automated blood pressure recorder (HEM-7310, Omron Healthcare Inc., Kyoto, Japan). Weight and height were measured thereafter. Body mass index (BMI) was calculated as the weight (in kilograms, kg) divided by the square of the height (in meters, m). Participants’ body composition were measured by a bioelectrical impedance analyzer (InBody 3.0, Biospace Co. Ltd., Tokyo, Japan). Waist circumference (WC) was measured at the level of the umbilicus to the nearest millimeter. The participants’ blood was then sampled for measurements of glucose, lipids, biochemistry, glycated hemoglobin (HbA_1c_), and AGEs.

A Jamar® hand dynamometer (Model 5030J1, Patterson Medical, Warrenville, IL, USA) was used to measure the grip strength of the dominant hand, with hand dominance determined by asking the participant which hand they favored for hammering. The dynamometer was set at the second or third handle position, whichever the participant stated was most comfortable. The participants standing with arms down by their side naturally and elbows in full extension, were instructed to squeeze the dynamometer using all of their strength. The best measurement from three trials was adopted for analysis.

A hand-held dynamometer (microFET2™, Hoggan Scientific, LLC, Salt Lake City, UT, USA) was used to measure the lower-limb muscle strength of the dominant leg (the preferred leg for kicking a ball). The participants were placed in the short sitting position with their knees and hips bent at right angles for testing their knee extensors and assumed a supine position for examination of the ankle dorsiflexors and plantar flexors. A trained examiner instructed the participants to actively apply force against the device while the examiner applied resistance. For each muscle group, the maximum reading of three measurements was recorded. Relative muscle strength was defined as muscle strength (in decinewtons, dN) divided by total muscle mass (in kg).

In addition, the patients received physical examination guided by the Michigan Neuropathy Screening Instrument (MNSI) for assessing diabetic peripheral neuropathy; abnormal findings were scored in accordance with the MNSI guidelines. The participants were next interviewed to collect information regarding their demographic characteristics and physical activity. To assess physical activity level, the participants reported the number of hours per day spent engaging in five designated levels of activity in a representative day over the previous 2 weeks. The five levels of activity were heavy (e.g., swimming), moderate (e.g., aerobic dancing), slight (e.g., casual walking), sedentary (sitting or standing), and basal (sleeping or lying down). A physical inactivity score, namely the ratio of sedentary hours over waking hours, was calculated using the formula reported previously.

Serum glucose, lipids, and biochemical parameters were measured using commercial assay kits (Roche Diagnostics, Basel, Switzerland) in an automatic blood chemistry analyzer (Roche-Hitachi 7180, Roche Diagnostics, Basel, Switzerland). Glycated hemoglobin was measured through capillary electrophoresis. Serum levels of AGEs were determined using OxiSelect™ Advanced Glycation End Product Competitive ELISA Kit (catalog number STA-817, Cell Biolabs, Inc., San Diego, CA, USA) in accordance with the manufacturer’s instructions. The concentrations of AGEs were calculated using the standard curve of AGE-bovine serum albumin.

### Statistical analysis

The study participants were divided into three groups on the basis of tertiles of serum levels of AGEs. Data are expressed as mean ± standard deviation or median (interquartile range) for continuous variables and number (percentage) for non-continuous variables. Because of the substantial skewing of the triglyceride and alanine aminotransferase values, logarithmic transformation was applied to reduce the skewness prior to analysis. One-way analysis of variance (with the Scheffe post hoc test) andχ^2^ tests were employed to compare the groups for the continuous and categorical variables, respectively.

The correlations between serum AGEs levels and relative muscle strength were examined using Pearson and partial correlation procedures. The following three models were constructed for partial correlation analysis: model 1, adjusted for age, sex, WC, physical inactivity score, and systolic blood pressure (SBP); model 2, adjusted for all parameters in model 1 and fasting glucose, serum creatinine (Cr), and high-density lipoprotein cholesterol (HDL-C); and model 3, adjusted for all parameters in model 2 and Michigan Neuropathy Screening Instrument-Physical Examination (MNSI-PE) score. Furthermore, we used multiple linear regression models to compare the degree to which AGEs, fasting glucose, and HbA_1c_ predicted relative muscle strength. The variables (in pairs or all three together) were entered into the models to examine the relative influences of these variables on relative muscle strength. A *P* value less than 0.05 indicated statistical significance. All analyses were performed using SPSS Statistics Program (version 22, IBM Corporation, Armonk, NY, USA).

## Results

A total of 152 participants were included in this study. The mean age of the participants was 71.1 ± 9.2 years, and 44.7% were men. The average BMI was 25.4 ± 3.5 kg/m^2^ (range, 18.0–35.0 kg/m^2^). The WC of the participants ranged widely, from 67.4 to 114.0 cm. The clinical characteristics of the participants based on tertiles of serum AGEs levels are listed in Table 1. The three groups of patients were comparable in terms of age, weight, BMI, WC, blood pressure, and physical inactivity score. Fasting glucose, HbA_1c_, and lipid profile were also balanced among the three groups. However, we observed a significant difference in MNSI-PE score among the three groups (*P* = 0.003).

**Table 1:** Clinical characteristics of study participants based on tertiles of serum levels of AGEs

	Low-tertile group	Mid-tertile group	High-tertile group	*P*
n	51	51	50	
Age (years)	69.1 ± 8.8	73.1 ± 9.6	71.0 ± 8.9	0.093
Men (n, %)	24 (47)	24 (47)	20 (40)	0.71
Weight (kg)	62.5 ± 10.1	65.5 ± 11.0	64.0 ± 12.0	0.40
BMI (kg/m^2^)	24.7 ± 3.1	25.7 ± 3.0	25.8 ± 4.2	0.24
WC (cm)	88.5 ± 7.9	92.7 ± 8.6	90.2 ± 11.1	0.079
Physical inactivity score	0.54 ± 0.23	0.60 ± 0.22	0.56 ± 0.21	0.42
SBP (mmHg)	136 ± 21	141 ± 17	144 ± 22	0.099
DBP (mmHg)	80 ± 11	80 ± 10	81 ± 10	0.83
MNSI-PE (score)	1.73 ± 1.34	2.54 ± 1.44	2.54 ± 1.29	0.003*
Fasting glucose (mg/dL)	126 ± 40	137 ± 40	128 ± 38	0.38
HbA_1c_ (%)	7.33 ± 1.09	7.79 ± 1.42	7.67 ± 1.43	0.19
LogALT	1.30 ± 0.23	1.30 ± 0.20	1.28 ± 0.18	0.79
Cr (mg/dL)	1.00 (0.75–1.00)	1.00 (0.80–1.09)	0.95 (0.82–1.18)	0.77
Total cholesterol (mg/dL)	154 ± 29	156 ± 29	156 ± 26	0.88
HDL-C (mg/dL)	50 ± 12	48 ± 13	47 ± 12	0.64
LogTG	2.01 ± 0.22	2.05 ± 0.22	2.06 ± 0.19	0.47
AGEs (ng/mL)	61.38 ± 13.95	97.66 ± 9.87	145.00 ± 32.92	< 0.0001*

Data are expressed as mean ± standard deviation or median (interquartile range) for continuous variables and number (percentage) for non-continuous variables. One-way analysis of variance was used to compare the three groups

*BMI* Body Mass Index, *WC* Waist Circumference, *SBP* Systolic Blood Pressure, *DBP* Diastolic Blood Pressure, *MNSI-PE* Michigan Neuropathy Screening Instrument Physical Examination score, *HbA*_*1c*_ glycosylated Hemoglobin, *ALT* Alanine aminotransferase, *LogALT* Logarithmic transformation of ALT (in U/L), *Cr* Creatinine, *HDL-C* High-density Lipoprotein Cholesterol, *LDL-C* Low-density Lipoprotein Cholesterol, *TG* Triglyceride, *LogTG* Logarithmic Transformation of TG (in mg/dL), *AGEs* Advanced Glycation End products

^*^P < 0.05

**Fig. 1:**
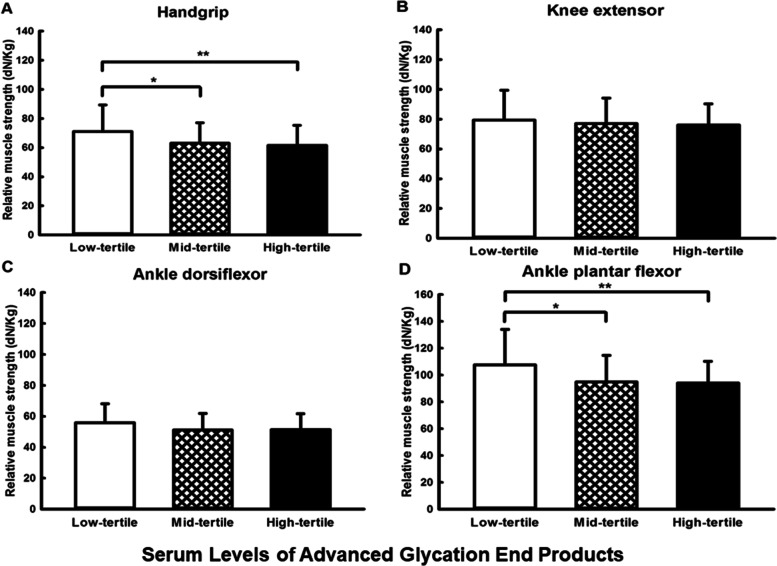
Relative muscle strength of handgrip, knee extensor, ankle dorsiflexor, and ankle plantar flexor, stratified on the basis of tertiles of serum levels of advanced glycation end products. *P < 0.05, **P < 0.01

Significant differences among the three groups were also noted in regards to relative handgrip strength (71.03 ± 18.22, 63.17 ± 13.82, and 61.47 ± 13.95 dN/kg in the low-tertile, mid-tertile, and high-tertile groups, respectively, *P* = 0.005; Fig. 1A). The relative muscle strength of the ankle plantar flexors was higher in the low-tertile group than in the mid-tertile and high-tertile groups (107.60 ± 26.53, 94.97 ± 19.72, and 94.18 ± 16.09 dN/kg in the low-tertile, mid-tertile, and high-tertile groups, respectively, *P* = 0.002; Fig. 1D). The differences in relative muscle strength of the ankle dorsiflexors were marginal among the three groups (*P* = 0.052; Fig. 1C). No significant differences were noted in relative muscle strength of the knee extensors among the three groups (*P* = 0.61; Fig. 1B).

The results of the Pearson correlation matrix are summarized in Table 2. Serum levels of AGEs were significantly correlated with SBP, MNSI-PE score, and relative muscle strength of the handgrip, ankle dorsiflexor, and ankle plantar flexor. Other clinical variables that significantly correlated with relative muscle strength included age, sex, WC, physical inactivity score, glucose, Cr, HDL-C, and MNSI-PE score (Table 2). After adjustment for multiple factors and MNSI-PE score, serum AGEs levels continued to be significantly negatively correlated with relative muscle strength of the upper and lower extremities (model 3, Table 3). Nevertheless, no correlation was observed between serum AGEs levels and relative muscle strength of the knee extensors, before or after adjustment (Table 2 and Table 3).

**Table 2:** Correlation matrix analysis of clinical characteristics, serum levels of AGEs, and relative muscle strength

	AGEs	Age	Male sex	WC	PI score	SBP	Glucose	HbA_1c_	LogALT	Cr	TC	HDL-C	LogTG	MNSI-PE	HG	KE	AD
Age	0.0030																
Male sex	-0.026	0.094															
WC	0.048	-0.025	0.13														
PI score	-0.013	0.30**	-0.062	0.052													
SBP	0.16*	0.21**	-0.16*	-0.064	0.11												
Glucose	0.024	-0.087	-0.096	0.056	0.065	0.0010											
HbA_1c_	0.13	-0.22**	-0.092	0.14	0.12	-0.051	0.54**										
LogALT	-0.22	-0.006	0.27**	0.13	-0.079	-0.11	0.20*	0.12									
Cr	0.087	0.24**	0.25**	0.15	0.20*	0.14	0.085	0.15	0.055								
TC	0.056	-0.33**	-0.18*	-0.055	-0.076	0.11	0.14	0.14	0.007	0.054							
HDL-C	-0.076	-0.095	-0.33**	-0.45**	-0.13	0.086	-0.070	-0.091	-0.20*	-0.22**	0.22**						
LogTG	0.091	-0.040	0.000	0.28**	0.17*	0.056	0.14	0.097	0.16*	0.18*	0.44**	-0.39*					
MNSI-PE	0.18*	0.26**	-0.048	0.21*	0.16*	0.057	0.27**	0.25**	-0.10	0.18*	-0.16	-0.14	0.000				
HG	-0.20*	-0.29**	0.23**	-0.34**	-0.19*	-0.10	-0.027	-0.057	0.15	-0.22**	0.055	0.18*	-0.15	-0.36**			
KE	-0.050	-0.16	-0.16	-0.25**	-0.11	0.042	-0.10	-0.073	0.038	-0.26**	0.13	0.26**	-0.077	-0.23**	0.45**		
AD	-0.19*	-0.19*	0.042	-0.37**	-0.059	< 0.001	-0.20*	-0.16	-0.008	-0.26**	0.079	0.21**	-0.10	-0.16	0.46**	0.46**	
AP	-0.22**	-0.18*	-0.25**	-0.40**	-0.031	0.079	-0.040	-0.11	-0.040	-0.23**	0.18*	0.31**	-0.16	-0.21**	0.41**	0.58**	0.56**

*AGEs* Advanced Glycation End products, *WC* Waist Circumference, *PI* Physical Inactivity, *SBP* Systolic Blood Pressure, *HbA*_*1c*_ glycosylated Hemoglobin, *LogALT* Logarithmic transformation of alanine aminotransferase, *Cr* Creatinine, *TC* Total Cholesterol, *HDL-C* High-density Lipoprotein Cholesterol, *LogTG* Logarithmic transformation of Triglyceride, *MNSI-PE* Michigan Neuropathy Screening Instrument Physical Examination score, *HG* relative muscle strength of Handgrip, *KE* relative muscle strength of Knee Extensor, *AD* relative muscle strength of Ankle Dorsiflexor, *AP* relative muscle strength of Ankle Plantar flexor

^*^P < 0.05, **P < 0.01 using Pearson correlation analysis

**Table 3:** Partial correlation coefficients between serum levels of advanced glycation end products and types of relative muscle strength

	Model 1		Model 2		Model 3	
	*r*	*P*	*r*	*P*	*r*	*P*
Handgrip	-0.21*	0.011	-0.19*	0.021	-0.17*	0.046
Knee extensor	-0.053	0.53	-0.031	0.72	-0.012	0.89
Ankle dorsiflexor	-0.20*	0.015	-0.19*	0.026	-0.20*	0.015
Ankle plantar flexor	-0.25**	0.002	-0.24**	0.004	-0.23**	0.006

Model 1: adjusted for age, sex, waist circumference, physical inactivity score, and systolic blood pressure

Model 2: adjusted for all parameters in model 1 and fasting glucose, serum creatinine, and high-density lipoprotein cholesterol

Model 3: adjusted for all parameters in model 2 and Michigan Neuropathy Screening Instrument-Physical Examination score

^*^P < 0.05, **P < 0.01

In the multiple linear regression analysis, serum AGEs levels remained significantly negatively associated with relative muscle strength (handgrip, ankle dorsiflexion, and ankle plantar flexion) when compared in pairs with fasting glucose or HbA_1c_ (Table 4). When all three parameters (serum AGEs levels, fasting glucose, and HbA_1c_) were entered into the models simultaneously, AGEs levels were the strongest variable that remained significantly associated with relative muscle strength of the upper and lower limbs (Table 4).

**Table 4:** Results of the multiple regression analyses with relative muscle strength of each muscle group as the dependent variable

Dependent variable	Variables entered	Estimate	SE	*P*
Handgrip	AGEs	-0.079	0.032	0.014*
	Fasting plasma glucose	-0.009	0.033	0.79
	AGEs	-0.078	0.032	0.016*
	HbA_1c_	-0.37	0.97	0.70
	Fasting plasma glucose	0.002	0.039	0.96
	HbA_1c_	-0.71	1.16	0.54
	AGEs	-0.078	0.032	0.017*
	Fasting plasma glucose	-0.003	0.039	0.94
	HbA_1c_	-0.32	1.15	0.78
Knee extensor	AGEs	-0.020	0.035	0.56
	Fasting plasma glucose	-0.045	0.036	0.21
	AGEs	-0.017	0.035	0.62
	HbA_1c_	-0.88	1.07	0.41
	Fasting plasma glucose	-0.040	0.042	0.35
	HbA_1c_	-0.32	1.25	0.80
	AGEs	-0.019	0.035	0.59
	Fasting plasma glucose	-0.041	0.043	0.34
	HbA_1c_	-0.23	1.26	0.86
Ankle dorsiflexor	AGEs	-0.053	0.022	0.018*
	Fasting plasma glucose	-0.057	0.023	0.013*
	AGEs	-0.049	0.023	0.030*
	HbA_1c_	-1.13	0.68	0.10
	Fasting plasma glucose	-0.049	0.027	0.078
	HbA_1c_	-0.56	0.81	0.49
	AGEs	-0.052	0.022	0.022*
	Fasting plasma glucose	-0.052	0.027	0.056
	HbA_1c_	-0.30	0.80	0.71
Ankle plantar flexor	AGEs	-0.12	0.044	0.007**
	Fasting plasma glucose	-0.019	0.045	0.67
	AGEs	-0.11	0.044	0.010*
	HbA_1c_	-1.36	1.33	0.31
	Fasting plasma glucose	0.015	0.054	0.78
	HbA_1c_	-2.05	1.60	0.20
	AGEs	-0.11	0.044	0.011*
	Fasting plasma glucose	0.007	0.053	0.89
	HbA_1c_	-1.48	1.58	0.35

*AGEs* Advanced Glycation End products, *HbA*_*1c*_ glycosylated Hemoglobin

^*^P < 0.05, **P < 0.01

## Discussion

In this study, we investigated serum levels of fluorescent AGEs in patients with type 2 diabetes. We revealed that high serum levels of AGEs were negatively associated with relative muscle strength in patients with type 2 diabetes after adjustment for clinical factors including diabetic peripheral neuropathy. Regarding their relative contribution, AGEs contributed to relative muscle strength decline more than fasting plasma glucose or HbA1c. These results indicate the potential of serum levels of AGEs as a surrogate marker of relative muscle strength in patients with type 2 diabetes.

Advanced glycation end products are a heterogeneous group of compounds with known deleterious effects, and AGEs can influence muscle function through various mechanisms in patients with diabetes [15]. Advanced glycation end products may cross-link extracellular matrix molecules, altering the signaling occurring between the matrix and cells and ultimately causing cellular dysfunction [9]. A study of aging animals demonstrated that the cross-linking of AGEs and collagen in muscle, tendons, and cartilage is associated with muscle stiffness, impaired muscle function, and accumulation of AGEs [16]. Advanced glycation end products may also play a role in muscle strength decline through the up-regulation of inflammation and endothelial dysfunction in the microcirculation of skeletal muscle through interaction with receptors for AGEs (RAGEs) [17]. Receptors for AGEs are cell surface receptors expressed in various tissues, including in vasculature, endothelium, smooth muscle cells, neural tissue, and mononuclear cells [17]. The activation of RAGEs induces the production of inflammatory cytokines and growth factors, which in turn drive oxidative stress, mitochondrial dysfunction, cell damage, and the overexpression of RAGEs in a well-known vicious circle [18].

Advanced glycation end products can be divided into nonfluorescent and fluorescent AGEs. Dalal et al. have proved the negative association between serum levels of carboxymethyl-lysine, a major nonfluorescent AGE, and grip strength in older community-dwelling adults [12]. Pentosidine is a fluorescent cross-linked AGEs formed in collagen [19]. Moriwaki et al. found that urinary pentosidine levels are negatively associated with grip strength and gait speed in middle-aged and elderly adults [20]. In this study, our findings suggest that fluorescent AGEs may play an independent role in the loss of muscle strength.

Advanced glycation end products are also key elements in the pathogenesis of diabetic neuropathy, directly by affecting structural and functional proteins and indirectly by activating RAGEs [21]. Muscle strength decline in patients with type 2 diabetes is related to the presence and severity of diabetic peripheral neuropathy [22]. Nomura et al. reported that middle-aged and older adult patients with diabetic peripheral neuropathy exhibited significantly reduced knee extension force between 10.9% and 16.5% compared with patients without diabetic peripheral neuropathy [23]. However, in our study, the inverse association between serum levels of AGEs and relative muscle strength remained significant after adjustment for clinical variables, including MNSI-PE score. Our results suggested the independent pathogenic role of AGEs in the gradual loss of muscle strength in patients with type 2 diabetes.

In the present study, Pearson correlation analysis indicated no significant association between relative muscle strength and glycemic control parameters (Table 2). Hiba et al. determined that the association between handgrip strength and glycemic control vanished after adjustment for clinical variables in middle-aged and older patients with diabetes [24]. However, other studies have reported that ineffective glycemic control was associated with lower relative muscle strength [25, 26]. The inconsistent results may be attributable to variations in the age range and BMI range of participants, physical activity level, and diabetic complications [22]. Further research is required to clarify the relationship between relative muscle strength and glycemic control.

The models of partial correlation analysis demonstrated the negative association between serum levels of AGEs and relative muscle strength of handgrip, ankle dorsiflexor, and ankle plantar flexor, but the association between serum levels of AGEs and relative muscle strength of the knee extensor was nonsignificant (Table 3). In patients with diabetes, the more distal ankle muscles decline in strength more than the muscles of the thigh [27]. The distal–proximal gradient of decreased muscle strength is also associated with diabetic peripheral neuropathy [28]. Our results indicated that the deleterious effects of AGEs are more intense in the distal part of limb muscle after adjustment for MNSI-PE. These results reveal that AGEs may play an independent role in the distal–proximal distribution of muscle strength decline in patients with type 2 diabetes.

In the current study, we demonstrated a positive association between serum levels of AGEs and SBP in patients with type 2 diabetes (Table 2). This finding is consistent with the findings of related research reporting that serum levels of AGEs correlate significantly with hypertension in patients with non-insulin dependent diabetes [29]. Advanced glycation end products can induce hypertension both through their altering of the arterial stiffness and their interaction with RAGEs leading to vascular dysfunction [30]. Several interventional trials employing AGEs as novel therapeutic strategies for the management of arterial stiffness and hypertension were proposed [31].

This study had several limitations. First, the analysis was based on cross-sectional data, and the observed associations cannot imply a causal relationship. Second, we measured circulating AGEs in serum, which were characterized by a heterogeneous class of compounds with diverse chemical structures. The association between specific AGEs and relative muscle strength in patients with type 2 diabetes can be further investigated. Lastly, serum levels of circulating AGEs did not sufficiently reflect the amount of AGEs in tissues, although related research reported a strong correlation between the levels of AGEs in serum and tissues [32]. Furthermore, the expression of RAGEs in muscle tissue was not assessed in this study. Further studies are necessary to determine the pathogenic role of AGEs in the development of diabetic muscular dysfunction in patients with type 2 diabetes.

## Conclusions

In conclusion, elevated serum levels of AGEs are inversely associated with relative muscle strength in patients with type 2 diabetes after adjustment for various clinical factors including diabetic peripheral neuropathy. Compared with fasting plasma glucose and HbA_1c_, serum level of AGEs is a more effective surrogate marker of relative muscle strength in patients with type 2 diabetes.

## Data Availability

The datasets generated and analyzed during the current study are not publicly available due to the datasets as part of an ongoing study but are available from the corresponding author on reasonable request.
